# Spending on Glucagon-Like Peptide-1 Receptor Agonists Among US Adults

**DOI:** 10.1001/jamanetworkopen.2025.2964

**Published:** 2025-04-02

**Authors:** Stavros Tsipas, Tamkeen Khan, Fleetwood Loustalot, Klodiana Myftari, Gregory Wozniak

**Affiliations:** 1 Improving Health Outcomes, American Medical Association, Chicago, Illinois; 2 Division for Heart Disease and Stroke Prevention, Centers for Disease Control and Prevention, Atlanta

## Abstract

This economic evaluation estimates annual total US spending on glucagon-like peptide-1 receptor agonists from 2018 to 2023.

## Introduction

In 2005, glucagon-like peptide-1 receptor agonists (GLP-1 RAs) were approved to treat type 2 diabetes. The GLP-1 RAs approved for type 2 diabetes before 2021 included dulaglutide (Trulicity), liraglutide (Saxenda and Victoza), exenatide (Bydueron and Byetta), and semaglutide (Ozempic and Rybelsus); and in 2022, a dual glucose-dependent insulinotropic polypeptide and GLP-1 RA, tirzepatide (Mounjaro). Two drugs were approved for weight loss among patients with at least 1 weight-related health comorbidity: liraglutide (Saxenda, 2014) and semaglutide (Wegovy, 2021).^1^ Lifestyle modification remains the cornerstone of chronic disease management, yet these new medications provide additional options for secondary prevention. This study analyzed annual total US spending on GLP-1 RAs from 2018 to 2023.

## Methods

The University of Illinois at Chicago institutional review board deemed this study exempt from review because it did not involve human participants. Spending for GLP-1 RA fills was obtained from Symphony Health data among adults aged 18 years or older. The data captured 85% of retail and 74% of mail order (ie, outpatient) prescription fills. Spending in this study represented the amounts directly spent by patients and insurers on GLP-1 RA fills at the point of sale and was adjusted for inflation using 2023 dollars.^2^ Analyses were conducted in 2024 using SAS version 9.4 (SAS Institute).

## Results

Total spending on GLP-1 RAs in the US increased by more than 500% from 2018 to 2023 (from $13.7 billion to $71.7 billion) (Table). Total spending grew by a mean of 34% per year from 2018 to 2022 and then grew by 62% from 2022 to 2023. The changes in spending between 2018 and 2023 varied by product. Spending for semaglutide (Ozempic) increased from $0.4 billion to $26.4 billion; and for dulaglutide (Trulicity), from $5.6 billion to $17.6 billion. Combined spending on liraglutide (Victoza) and exenatide (Bydureon, Byetta) decreased from $7.1 billion to $3.1 billion, whereas spending on liraglutide (Saxenda) increased slightly from $0.6 to $0.9 billion. Spending on semaglutide (Rybelsus), semagalutide (Wegovy), and tirzepatide (Mounjaro) has only increased since their introduction. From 2018 to 2023, the share of total GLP-1 RA spending for dulaglutide (Trulicity) declined from 41% to 25% and the combined share of liraglutide (Victoza) and exenatide (Bydureon, Byetta) declined from 52% to 4% (Figure). By 2023, semaglutide (Ozempic, Rybelsus, and Wegovy) and tirzepatide (Mounjaro) made up 70% of all spending. The 2 products released since 2021, Mounjaro and Wegovy, had 17% and 10% shares, respectively. In 2023, products with an approved indication of type 2 diabetes accounted for 89% of all spending while products with an approved indication of obesity accounted for 11% of all spending.

**Table.** Total Spending on GLP-1 RAs by Product^a^

GLP-1 RA	Spending, $ (billions)					
	2018	2019	2020	2021	2022	2023
Indicated for type 2 diabetes						
Liraglutide (Victoza)	5.44	5.46	5.05	4.27	3.32	2.62
Exenatide (Bydureon, Byetta)	1.66	1.61	1.49	1.06	0.73	0.50
Dulaglutide (Trulicity)	5.60	7.99	10.63	13.76	16.54	17.57
Semaglutide (Ozempic)	0.41	2.74	6.12	9.56	15.57	26.42
Semaglutide (Rybelsus)	NA	0.02	0.58	1.67	2.87	4.25
Tirzepatide (Mounjaro)	NA	NA	NA	NA	2.51	12.42
Type 2 diabetes subtotal	13.11	17.82	23.87	30.32	41.54	63.78
Indicated for obesity						
Liraglutide (Saxenda)	0.56	0.69	0.79	0.89	1.05	0.89
Semaglutide (Wegovy)	NA	NA	NA	0.58	1.58	6.99
Obesity subtotal	0.56	0.69	0.79	1.47	2.63	7.88
Total	13.66	18.51	24.67	31.79	44.18	71.66

Abbreviations: GLP-1 RA, glucagon-like peptide-1 receptor agonist; NA, not applicable.

^a^ Spending based on transacted prices and adjusted for inflation using constant 2023 US dollars. Tirzepatide (Zepbound) was approved for use among adults with obesity or overweight and at least 1 weight-related condition on November 8, 2023, and was not included in this analysis.

**Figure.**
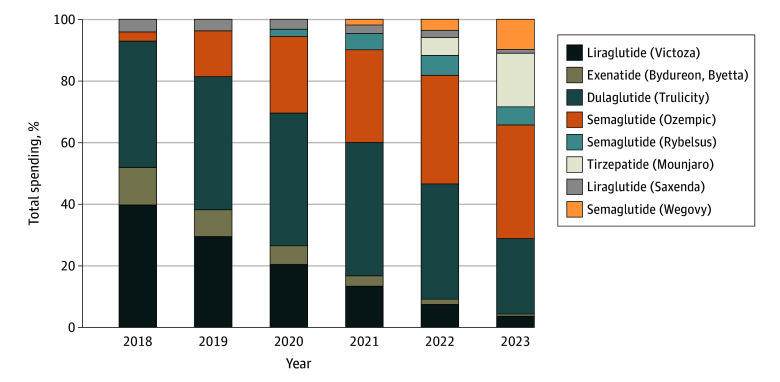
Percentage of Total Spending by Glucagon-Like Peptide-1 Receptor Agonist Product Product shares sum to 100%.

## Discussion

Spending on GLP-1 RAs increased from 2018 to 2023, with the largest growth rates from 2022 to 2023. Although spending for certain GLP-1 RAs increased substantially, spending declined for others. This study estimated that more than $71 billion was spent on GLP-1 RAs and more than $50 billion on a product based on either semaglutide or tirzepatide molecules.

Prescription drug spending estimates are complex and frequently lack transparency at the product level.^3^ Dataset limitations excluded sales at compounding pharmacies, prescription indication, and prevented adjustment for rebates, discounts, or price concessions. These vary across manufacturers and can be substantial, especially on newly released products. For example, manufacturer discounts for GLP-1 RAs prior to the introduction of tirzepatide have been estimated to be as much as approximately 40% to 60%.^4^ Although this study’s results suggest dramatic increases in net spending on GLP-1 RAs, the actual spending is likely somewhat lower than our estimates.

The expanded indications and coverage determination in future years could continue to drive demand and spending. Challenges in long-term adherence,^5^ spending in competition with other health care costs, lack of price transparency, and the integration of new products and indications will continue to drive demand for ongoing research in this area.

## Notes

Supplement.
Data Sharing Statement
