# Supplementary material for: Spending on Glucagon-Like Peptide-1 Receptor Agonists Among US Adults
Source: JAMA Netw Open. 2025 Apr 2;8(4):e252964. doi: 10.1001/jamanetworkopen.2025.2964 (PMC11966331; doi:10.1001/jamanetworkopen.2025.2964)
Supplement: Supplement. — Data Sharing Statement [file jamanetwopen-e252964-s001.pdf]

Data Sharing Statement

Tsipas. Spending on Glucagon-Like Peptide-1 Receptor Agonists Among US Adults. *JAMA Netw Open*. Published April 02, 2025. doi:10.1001/jamanetworkopen.2025.2964

Data
Data available: No

Additional Information
Explanation for why data not available: We licensed the data from Symphony Health. As part of our license, we cannot share the data with others. If desired, other researchers can license the data from Symphony Health. Symphony Health Solutions Integrated Dataverse® data available: https://www.iconplc.com/solutions/real-world-intelligence/symphony-health
